# Characterization of the complete chloroplast genome of *Engelhardia roxburghiana* (Juglandaceae)

**DOI:** 10.1080/23802359.2019.1700197

**Published:** 2019-12-13

**Authors:** Li-Zhen Ling, Shu-Dong Zhang

**Affiliations:** School of Biological Sciences and Technology, Liupanshui Normal University, Liupanshui, China

**Keywords:** Chloroplast genome, *Engelhardia roxburghiana*, Juglandaceae, phylogenetic analysis

## Abstract

*Engelhardia roxburghiana* is a traditional Chinese medicine in the Juglandaceae family. In this study, the complete chloroplast (cp) genome sequence of *E. roxburghiana* was reported and characterized. The cp genome is 161,550 bp in length and contains a pair of inverted repeats (IRs, 25,862 bp) separated by a large (90,937 bp) and small (18,889 bp) single-copy regions. A total of 113 unique genes were predicted, including 79 protein-coding genes, 30 tRNA genes and 4 rRNA genes. The phylogenetic analysis suggested that *E. roxburghiana* is resolved as a sister group to the clade of subfam. Juglandoideae.

*Engelhardia roxburghiana* Wallich is an evergreen to briefly deciduous tree in the Juglandaceae family. It is mainly distributed in China, Thailand, Vietnam and other Asia regions (Lu et al. 1999). The bark and leaves of this species contain active pharmaceutical ingredients, which may contribute to inhibit aldose reductase and sorbitol, enhance the vanadate-stimulated release of lipoprotein lipase, anti-inflammatory, antioxidant and antitubercular effects (Hong et al. 2012; Huang et al. 2011; Wu et al. 2007; Xin et al. 2012). The bark appearance of *E. roxburghiana* is very similar to that of *Magnolia officinalis* Rehder & E. H. Wilson, another traditional Chinese medicine and endangered species (Cao et al. 2002). Usually, the dried bark of *E. roxburghiana* has been used as the confusable bark of *M. officinalis*. To avoid the misuse and accurate identification of the bark of *E. roxburghiana*, we sequenced and analyzed the complete chloroplast (cp) genome of this species using high-throughput sequencing technology.

The total genomic DNA was extracted from fresh leaves collected from Kunming (Yunnan, China, N25°08’11”, E102°44’23”, 1,950 m) and voucher specimen (lpssy0310) was deposited at the herbarium of the Liupanshui Normal University (LPSNU). High-throughput sequencing was performed using the Illumina HiSeq 2500 platform. Approximately 2 Gb raw data were used to *de novo* assemble the complete cp genome using SPAdes (Bankevich et al. 2012). All genes of the genome were annotated using PGA (Qu et al. 2019).

The complete *E. roxburghiana* cp genome (accession number MN652922) was 161,550 bp in length, which is little longer than those of several species of *Juglans* (Dong et al. 2017). The cp genome possessed the typical quadripartite structure of angiosperms, consisting of a pair of the inverted repeat (IR) regions of 25,862 bp each, a large single copy (LSC) region of 90,937 bp, and a small single copy (SSC) region of 18,889 bp. The cp genome showed the GC content of 35.9% and contained 113 unique genes, including 79 protein-coding genes, 30 transfer RNA (tRNA) genes, and 4 ribosomal RNA (rRNA) genes. Of them, 15 distinct genes (*atpF*, *ndhA*, *ndhB*, *petB*, *petD*, *rpl16*, *rpl2*, *rpoC1*, *rps16*, *trnA-UGC*, *trnG-GCC*, *trnl-GAU*, *trnK-UUU*, *trnL-UAA* and *trnV-UAC*) contained one intron and three genes (*clpP*, *rps12* and *ycf3*) had two introns.

The Juglandaceae family comprises two subfamilies (Engelhardioideae and Juglandoideae) (Manos and Stone 2001) and seven genera (*Annamocarya*, *Carya*, *Cyclocarya*, *Engelhardtia*, *Juglans*, *Platycarya* and *Pterocarya*) (Lu et al. 1999). To understand the phylogenetic position of *E. roxburghiana* within Juglandaceae, we downloaded the complete cp genomes of 15 species from the NCBI GenBank database, including 14 species in Juglandaceae and one species (*Rhoiptelea chiliantha*) in Rhoipteleaceae as outgroup. RAxML v8.2.4 (Stamatakis 2014) and MrBayes v3.2.3 (Ronquist and Huelsenbeck 2003) were used to perform maximum likelihood (ML) and Bayesian inference (BI) analyses. The phylogenetic tree showed that there were two major clades in Juglandaceae (Figure 1), which is in agreement with the two subfamily classifications (Manos and Stone 2001). *Engelhardtia roxburghiana*, the member of the Engelhardioideae, formed a sister clade with the Juglandoideae (Figure 1).

**Figure 1.**
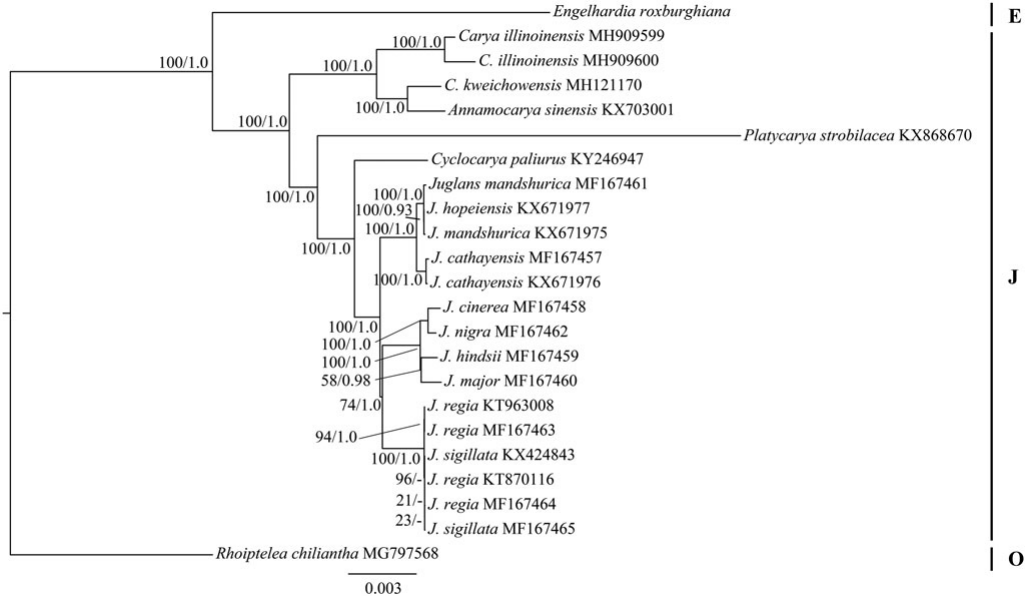
The maximum likelihood (ML) tree of 15 species from the family Juglandaceae inferred from the complete chloroplast genome sequences. Numbers at nodes correspond to ML bootstrap percentages (1,000 replicates) and Bayesian inference (BI) posterior probabilities. E: Engelhardioideae, J: Juglandoideae, O: outgroup.
